# Construct validity of the Suboptimal Health Status Questionnaire-25 in a Ghanaian population

**DOI:** 10.1186/s12955-021-01810-z

**Published:** 2021-07-19

**Authors:** Eric Adua, Ebenezer Afrifa-Yamoah, Kwasi Frimpong, Esther Adama, Shantha P. Karthigesu, Enoch Odame Anto, Emmanuel Aboagye, Yuxiang Yan, Youxin Wang, Xuerui Tan, Wei Wang

**Affiliations:** 1 Center for Precision Health, Edith Cowan University, 270 Joondalup Drive, Joondalup, WA Australia; 2 Shantou University of Medical College, Shantou, China; 3 Department of Biochemistry, Kwame Nkrumah University of Science and Technology, Kumasi, Ghana; 4 School of Science, Edith Cowan University, 270 Joondalup Drive, Joondalup, WA Australia; 5 Ghana Institute of Management and Public Administration, Accra, Ghana; 6 School of Nursing and Midwifery, Edith Cowan University, 270 Joondalup Drive, Joondalup, WA Australia; 7 Department of Medical Diagnostics, Faculty of Allied Health Sciences, Kwame Nkrumah University of Science and Technology, Kumasi, Ghana; 8 Institute of Environmental Medicine, Karolinska Institute, Nobels Väg 13, 17177 Stockholm, Sweden; 9 Beijing Key Laboratory of Clinical Epidemiology, School of Public Health, Capital Medical University, Beijing, 100069 China

**Keywords:** Factor analysis, Suboptimal Health Status Questionnaire, Construct validity, Structural equation modelling

## Abstract

**Background:**

The Suboptimal Health Status Questionnaire-25 (SHS-Q-25) developed to measure Suboptimal Health Status has been used worldwide, but its construct validity has only been tested in the Chinese population. Applying Structural Equation Modelling, we investigate aspects of the construct validity of the SHS-Q-25 to determine the interactions between SHS subscales in a Ghanaian population.

**Methods:**

The study involved healthy Ghanaian participants (n = 263; aged 20–80 years; 63% female), who responded to the SHSQ-25. In an exploratory factor and parallel analysis, the study extracted a new domain structure and compared to the established five-domain structure of SHSQ-25. A confirmatory factor analysis (CFA) was conducted and the fit of the model further discussed. Invariance analysis was carried out to establish the consistency of the instrument across multi-groups.

**Results:**

The extracted domains were reliable with Cronbach’s $\alpha$ of 0.846, 0.820 and 0.864 respectively, for fatigue, immune-cardiovascular and cognitive. The CFA revealed that the model fit indices were excellent $\left( {{\text{RMSEA}} = 0.049~ < ~0.08,\,{\text{CFI}} = 0.903 > 0.9,\,{\text{GFI}} = 0.880 < 0.9,\,{\text{TLI}} = 0.907 > 0.9} \right)$. The fit indices for the three-domain model were statistically superior to the five-domain model. There were, however, issues of insufficient discriminant validity as some average variance extracts were smaller than the corresponding maximum shared variance. The three-domain model was invariant for all constrained aspects of the structural model across age, which is an important risk factor for most chronic diseases.

**Conclusion:**

The validity tests suggest that the SHS-Q25 can measure SHS in a Ghanaian population. It can be recommended as a screening tool to early detect chronic diseases especially in developing countries where access to facilities is diminished.

## Background

Since the current testing and treatment of symptomatic chronic disease is considered a delayed response, it has become generally accepted that early detection provides better treatment options and ensures better quality of life [1, 2]. Targeting at-risk individuals is critical, as they can be counselled and provided with prophylactic therapies that can potentially reduce or prevent their risk [1, 2]. To achieve this, researchers have resorted to using health assessment or screening instruments or tools, usually subjective questionnaires, to measure individual’s dietary habits [3], physical activities [4] and work productivity [5]. Although reliance and usage of such questionnaires have promoted clinical diagnosis and lifestyle modifications, their clinical relevance has been eclipsed by the cumbersome and ambiguous nature of some of the questions, the time required to complete the questionnaire and the challenges of interpreting the results. For these reasons, a more streamlined and targeted instrument is required.

Over the last few years, some advances in research have been made in the design of robust screening instruments, giving rise to the widely used Suboptimal Health Status Questionnaire-25 (SHSQ-25) [6–8]. Popularly articulated and operationalised in 2009, the SHSQ-25 has had leverage over the existing instruments due to its simplicity, clearly described questions and the simple scoring system [9, 10]. Importantly, it encapsulates questions that comprehensively capture multiple indicators of good health, including fatigue, the cardiovascular system, the immune system, mental status and the digestive tract [6, 9, 11]. When completed, SHSQ-25 can reveal individuals who may be experiencing poor health that cannot be traced to a particular disease, referred to as Suboptimal Health Status (SHS) [6, 10–12].

SHS represents an intervening state, prior to chronic disease, that is often hallmarked by a lack of vitality, body weakness and loss of appetite [9, 13]. It has become a major public health concern worldwide, as its link to different chronic diseases traverses across multiple populations [6, 8, 9, 12, 14, 15]. Among the mainland Chinese, SHS was found to be associated with commonly known cardiovascular risk factors including psychosocial stress [10, 16], physical inactivity, increased blood pressure, plasma glucose and abnormal lipid profiles [9, 11]. In a Russian population, SHS was associated with endothelial dysfunction [7] and among Ghanaians, it was a precursor to type II diabetes mellitus [7, 12]. Following their analyses of hematobiochemical, sociodemographic and clinical data, Anto et al. [15] indicated the presence of SHS before preeclampsia among pregnant women in Ghana [15]. Among Chinese youths, SHS was associated with altered intestinal microbiota [17]. Furthermore, its association with objective markers including plasma cortisol, mRNA expression of glucocorticoid receptor α/β [10], plasma metabolites [13], N-glycosylation profiles [14], telomere length [18] and oxidative stress [19] as well as angiogenic growth mediators [19] have been reported.

Despite its widespread applications, studies that explore the psychometric properties of the SHSQ-25 are inadequate. The first and only study to date, that tested the validity and reliability of SHSQ25 was conducted in a Chinese population [6]. In this study, they applied statistical methods such as test–retest reliability, internal consistency, convergent validity, along with factor and exploratory analysis to show that SHS-Q25 is capable of detecting SHS [6]. Although this study highlights some psychometric testing, its construct validity has not been evaluated outside of China. This information is critically important because the relative validity and reliability of tools may not be the same in different populations, especially an African population such as Ghana.

On the one hand, Ghanaians in urban cities share similarities with Chinese in terms of urbanisation, increased work stress and pressures from home [20, 21]. As such, the prevalence of SHS might be the same in both countries. On the other hand, Ghanaians have different genetic composition, varied job types, climatic conditions, different cultures and dietary differences that may make them susceptible to SHS or even a chronic disease [20]. In addition, the extent of correlations between the metrics or components in each of the five domains have not been properly reported. Taken together, these constitute a significant research interstice and provide a justification for this present study.

Following on our previous studies [6, 8, 10, 22], with the goal of exploring the cross-national comparability of SHSQ-25 and emphasising on the robustness of the SHSQ-25, this current study aims to investigate the aspects of construct validity of the SHS-Q25 by applying a Structural Equation Model (SEM) to determine the association between SHS subscales in a Ghanaian population. Understanding the nature of relationships between domains and determining the scores for the various domains will guide intervention practices.

### Instrument development, study design and methods

**Fig. 1:**
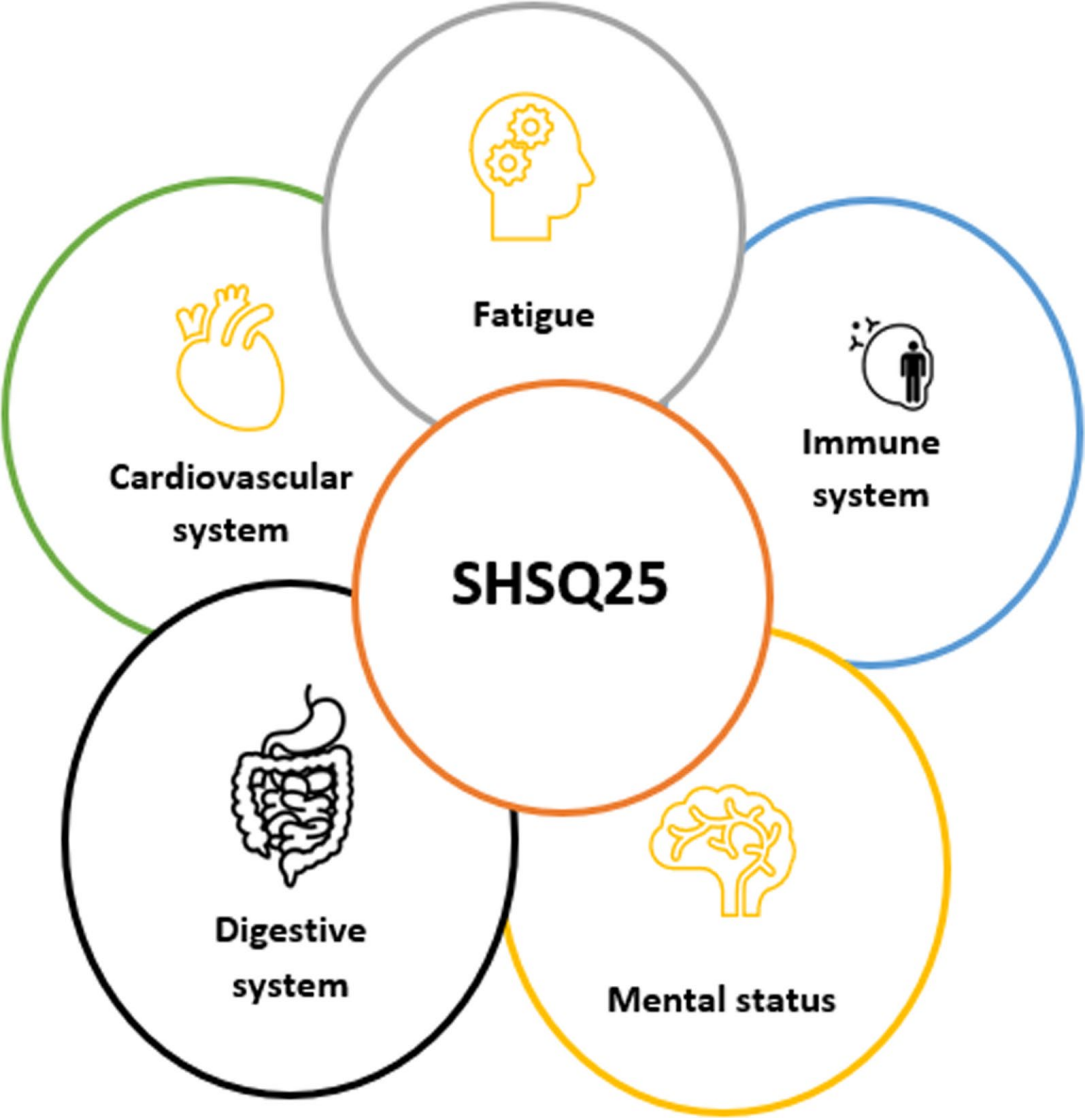
Conceptual framework of Suboptimal Health Status Questionnaire-25. The instrument provides a holistic assessment of human health based on five health domains: cardiovascular system, digestive, immune, mental health, and fatigue. When completed, the health care provider can assess the health status of an individual long before a chronic disease onset and thereby facilitating preventative healthcare

The quality and level of health as defined by the World Health Organisation (WHO), is far-reaching for most people. Increasingly, people report feeling unwell, but their malaise cannot be traced to a particular diagnosable disease. Previous research shows that individuals with sub-optimal health status (SHS) suffer from symptoms such as chronic fatigue, headaches, dizziness, depression, anxiety, nonspecific pain and functional disorders [6–10]. These individuals may present with reduced organ function and physical functionality, energy loss, low cognitive and emotional performance or even decline in social functioning [6–10]. Altogether, these abnormal symptoms can worsen into major cardiovascular, immune, digestive, psychological disorders. The SHSQ-25 questionnaire composed of 25 questions, was developed to multidimensionally capture health constructs that could reveal individuals experiencing poor health status. The instrument was derived from five health domains: immune system (3 items), mental health (7 items), fatigue (9 items), digestive system (3 items), and cardiovascular system (3 items) (Fig. 1). The questionnaire is the outcome of extensive literature search, taking clues from established instruments such as Traditional Chinese Medicine Syndrome Questionnaire of Subhealth Status” and the Cornell Medical Index [6–10]. It was then pretested in focus groups to determine the relevance and the clarity of each item. All ambiguous questions and redundant questions were removed to ensure content validity. Using a 5-point Likert type scale, participants indicated their health status by selecting the following options (1) never or almost never, (2) occasionally, (3) often, (4) very often and (5) always [6–10].

In a cross-sectional study, 263 apparently healthy individuals were recruited from the Kumasi Metropolis of Ghana using convenient sampling technique. The SHSQ-25 was used to measure SHS for all participants. The study excluded all participants with known clinical conditions such as hypertension, respiratory, genitourinary and haematological problems. Participants aged 18–80 years were included. Because a proportion of the participants barely understood the English, we applied both forward and reverse translational methods. First, we engaged health professionals who understood the local language and the terminologies in the questionnaire to translate the questions in way that achieved conceptual equivalence with the original questionnaire. They ensured that medical jargons, colloquialism and technical terms and literal translation were removed. After this, a panel of research assistants, experts in questionnaire development and translation were involved to resolve any inadequate expressions or discrepancies in the translation. Then, a back translation was done where the translated instrument was translated back by an expert in to confirm conceptual equivalence. Following this, a pre-testing of the instrument on a target population was performed. All interviews were conducted by experienced interviewers. This study is part of a larger study where 523 participants were recruited [12]. After performing a power analysis using G*Power software version 3.1.9.2, the sample size reached a statistical power of 80%, with an effect size of 0.5 at an alpha level of 0.01.

### Clinical data

Systolic and diastolic blood pressures (SBP and DBP) were measured with a sphygmomanometer. Using a standard stadiometer (SECA, Hamburg, Germany), we measured the height (cm) and weight (kg). From these, body mass index (BMI) was calculated using the formula BMI = weight (kg)/height (m)^2^. Tape measure was used to measure the waist and hip circumference. Prior to detecting fasting plasma glucose (FPG), we collected blood samples from the antecubital vein into fluoride oxalate coated tubes. Levels of sugar were detected on an automated chemistry analyser (Roche Diagnostics, COBAS INTEGRA 400 Plus, USA). The reason for the inclusion of the clinical data is to objectively reveal the health state of the participants.

### Statistical analyses

The appropriateness of the data was assessed using the Kaiser–Meyer–Olkin (KMO) statistic and the Bartlett’s test of sphericity. The reliability of the items in each domain was assessed by Cronbach’s alpha. The Structural Equation Model (SEM) was used in a confirmatory factor analysis (CFA). The goodness-of-fit of models were assessed using appropriate indices such as comparative fit index (CFI), root mean square error of approximation (RMSEA), goodness-of-fit index (GFI), and Tucker–Lewis Index (TLI). We further calculated the composite reliability (CR) statistics to establish the construct validity or otherwise of the SHSQ-25 instrument. The average variance extract (AVE) and maximum shared variance (MSV) were used to assess the convergent and discriminant validity of the instrument. The results reached statistical significance at an alpha level of 0.05. We investigated new domain structure of the SHSQ-25 instrument using parallel analysis. Invariance analysis was performed to assess the specification equivalence across various groupings in the dataset, namely; gender (male and female), age group (subjects above average age, subjects below average age) and marital status (married and not married) for unconstrained models, models constrained on the factor loadings, models constrained on the structural covariance loadings and models constrained on the residual covariance loadings. IBM AMOS 25 was used for the analysis.

## Results

The dataset consisted of 263 healthy Ghanaian individuals, male (n = 96) and female (n = 167), aged between 20 and 80 years (M = 51.32, SD = 12.25). In general, females had increased BMI (27.30 ± 5.24, *p* < 0.001) and waist-to height ratio (WHtR) (0.58 ± 0.08, *p* < 0.001) compared to males. However, there was no statistically significant difference in FPG, and SBP between males and females. Most males and females had some form of education and employment (Table 1).

**Table 1:** Characteristics of study participants stratified by gender

Variable	Male (n = 96)	Female (n = 167)	Statistic	*p *value
Age (years) (n = 262)	51.95 ± 11.99	50.97 ± 12.45	7742.5^u^	0.7027
*BMI*			43.149^	** < 0.001**
Underweight	7 (7.4)	5 (3.0)		
Normal weight	58 (61.1)	50 (29.9)		
Overweight	28 (29.5)	58 (34.7)		
Obese	2 (2.1)	54 (32.3)		
*Education*			24.47^	** < 0.001**
Tertiary	25 (26.3)	10 (6)		
Senior high school	25 (26.3)	57 (34.3)		
Junior high school	33 (34.7)	58 (34.9)		
Lower primary	6 (6.3)	25 (15.1)		
No formal education	6 (6.3)	16 (9.6)		
*Occupation*			19.53^	**0.0020**
Employed	73 (76.8)	110 (66.3)		
Retired	11 (11.6)	10 (6.0)		
Keeping house	1 (1.1)	16 (9.6)		
Unemployed	0 (0)	15 (9)		
Informal	10 (10.5)	15 (9)		
*T2DM history*			2.55^	0.2790
Yes	39 (41.1)	78 (47.3)		
*Clinical data*				
WHtR	0.51 ± 0.06	0.58 ± 0.08	3833^u^	** < 0.001**
BMI (kg/m^2^)	23.15 ± 3.51	27.30 ± 5.24	4179.5^u^	** < 0.001**
SBP (mmHg)	146.99 ± 26.96	141.58 ± 22.18	7248^u^	0.2230
DBP (mmHg)	81.94 ± 15.71	85.67 ± 13.46	6670.5^u^	**0.0281**
FPG (mmol/l)	5.73 ± 0.75	5.87 ± 0.99	7306.5^u^	0.3329

Data presented as Mean ± SD and n (%). ^χ^2^ test of independence, ^u^Mann Whitney U tests. Tests of significance were two tailed and bolded (*p < 0.05)

### Baseline results: conceptual model for the SHSQ-25 instrument

**Fig. 2:**
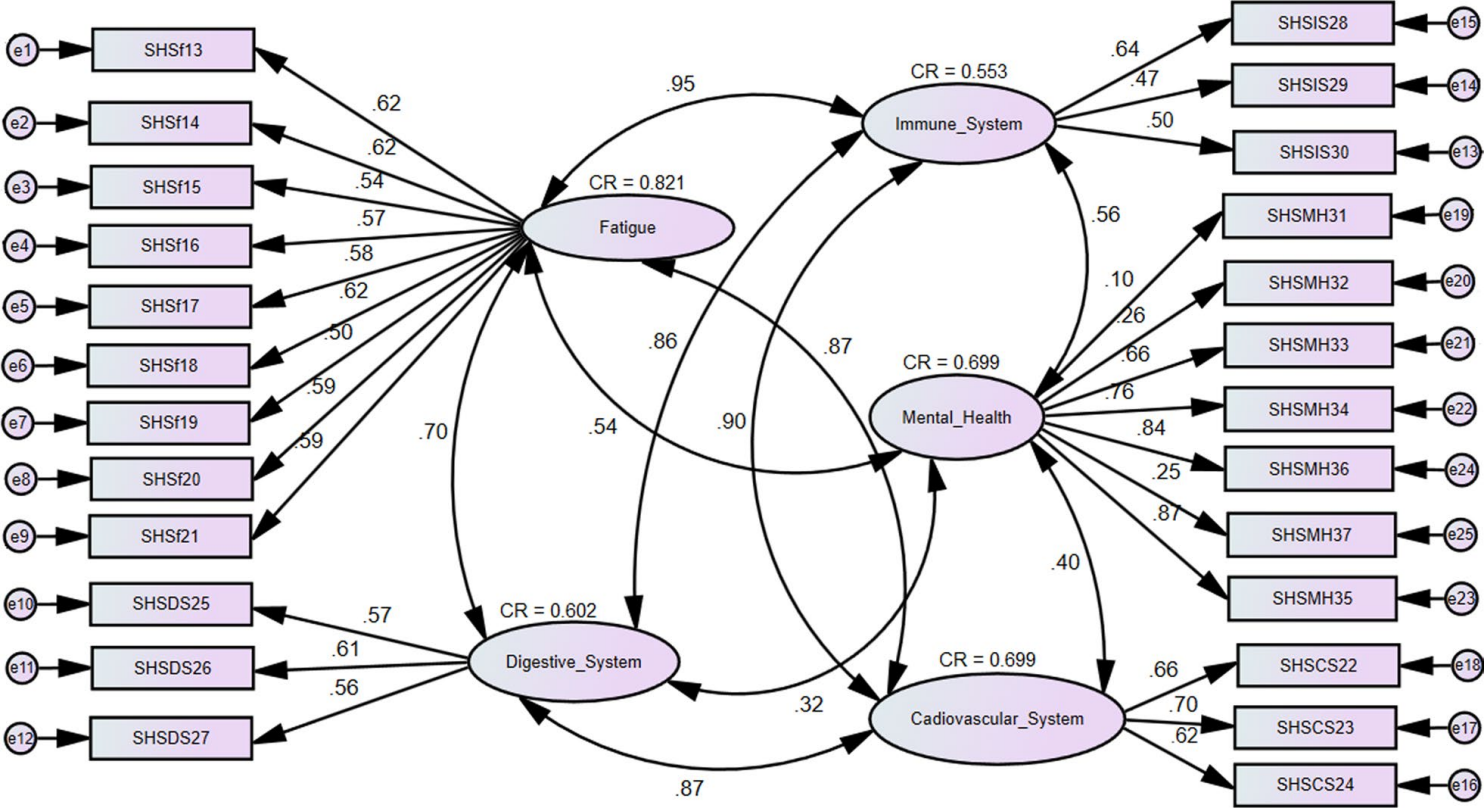
Confirmatory factor model showing the standardized factor loadings for the five-domain structure of the SHSQ-25 instrument. Each of the five domains showed a good-moderate reliability; immune system (IS) (0.553); fatigue (F) (0.821); digestive system (DS) (0.602); mental health (MH) (0.699) and cardiovascular system (CS) (0.699)

Figure 2 presents the conceptual model for the SHSQ-25 instrument showing the measures of the relationship between the latent variables and questionnaire items. The overall fit of the model was adequate with ${\text{RMSEA}}~ = 0.098 > 0.05,\,{\text{CFI}} = 0.809 < 0.9,\,{\text{GFI}} = 0.781 < 0.9,\,{\text{TLI}} = 0.791 < 0.9$. There were very high correlations among the latent variables, which affected the discriminant validity of the domains of the instrument. For instance, the correlational values are fatigue and immune system (R = 0.949), immune system and cardiovascular system (R = 0.904), fatigue and cardiovascular system (R = 0.873), digestive system and cardiovascular system (R = 0.87), and digestive system and fatigue (R = 0.70). However, there was a relatively low correlation between immune system and mental health (R = 0.56), and mental health and cardiovascular system (R = 0.40). In terms of discriminant validity, except for mental health (AVE = 0.372, MSV = 0.317), the four other domains did not achieve satisfactory measure as the AVE scores were less than the MSV scores. There were also convergent validity issues as the AVE for the latent variables were below the 0.5 threshold: fatigue (0.339), immune system (0.296), cardiovascular system (0.437), mental health (0.372) and digestive system (0.335). The composite reliability (CR) measures for the latent variables were below 0.7 except for fatigue and mental health (Fig. 2).

### New domain extraction

**Fig. 3:**
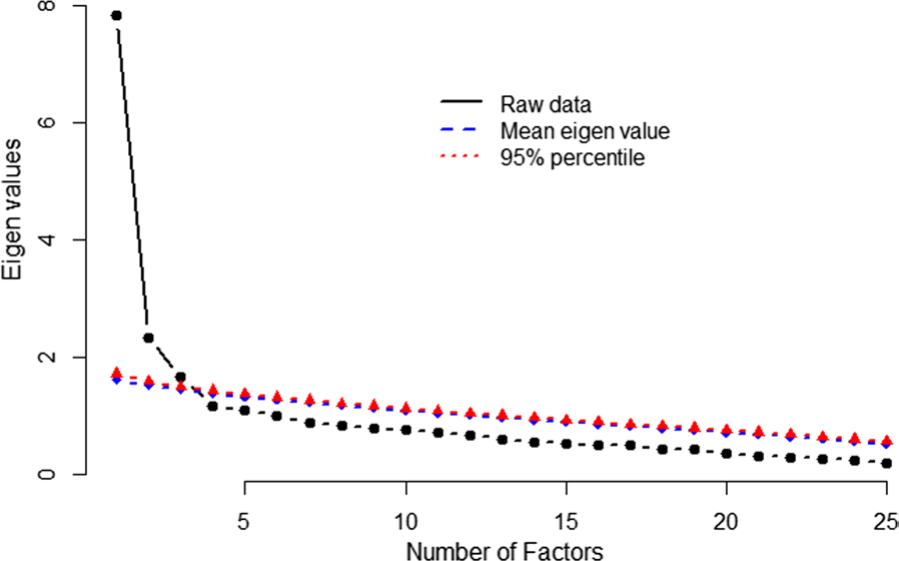
Scree plot and parallel analysis. The scree plot line (in black) indicates the rate of decline in the eigen values to establish the number of factors deemed as adequate. The parallel analysis presents the mean eigen value (in blue) and the 95th percentile value (in red)

The sample adequacy was established based on KMO = 0.889. The Bartlett’s test of sphericity produced a *p *value < 0.001, indicating that the dataset diverges significantly from the identity matrix, making the data set suitable for data reduction. A parallel analysis revealed that a three- factor structure was more appropriate (Fig. 3).

The internal consistency of the domains was assessed using the Cronbach’s α and item-delete Cronbach’s α. The internal consistency was good with Cronbach’s α statistics lying between $0.7 \le \alpha < 0.9$. Table 2 presents the Cronbach’s α and the item-delete Cronbach’s α for the three-domain.

**Table 2:** Internal consistency of the three-factors structure

Domain/Cronbach’s α	Label	Item (each question is preceded by *in the past 3 months*)	Cronbach’s α if item is deleted
Fatigue (0.861)	SHSf13	How often were you exhausted without greatly increasing your physical activity?	0.839
	SHSf14	How often did you have fatigue which could not be substantially alleviated by rest?	0.841
	SHSf15	How often were you lethargic in your daily life?	0.847
	SHSf16	How often did you suffer from headaches?	0.855
	SHSf18	How often did your eyes ache or feel tired?	0.852
	SHSf19	How often did your muscles or joints feel stiff?	0.846
	SHSf20	How often did you have pain in your shoulders/neck/back?	0.846
	SHSf21	How often did you have a heavy feeling in your legs when walking?	0.847
	SHSCS22	How often did you feel out of breath while resting?	0.852
	SHSCS28	How often did you have difficulty tolerating hot and cold temperatures?	0.851
	SHSf17	How often did you suffer from dizziness?	0.809
Immuno-cardiovascular (0.821)	SHSCS23	How often did you suffer from chest congestion?	0.792
	SHSSC24	How often were you bothered by heart palpitations?	0.806
	SHSDS25	How often was your appetite poor?	0.808
	SHSDS26	How often did you suffer from heartburn?	0.804
	SHSDS27	How often did you suffer from nausea?	0.805
	SHSIS29	How often did you catch a cold?	0.808
	SHSIS30	How often did you suffer from a sore throat?	0.801
	SHSMS31	How often did you have difficulty falling asleep?	0.817
	SHSMH37	How often were you troubled by waking up during the night?	0.813
	SHSMH37	How often did you feel nervous or jittery	0.803
Cognition (0.853)	SHSMH33	How often did you have trouble with your short-term memory?	0.863
	SHSMH34	How often did you did you have difficulty responding to situations quickly or making decisions?	0.802
	SHSMH35	How often did you have difficulty concentrating?	0.790
	SHSMH36	How often were you distracted for no reason?	0.808

**Fig. 4:**
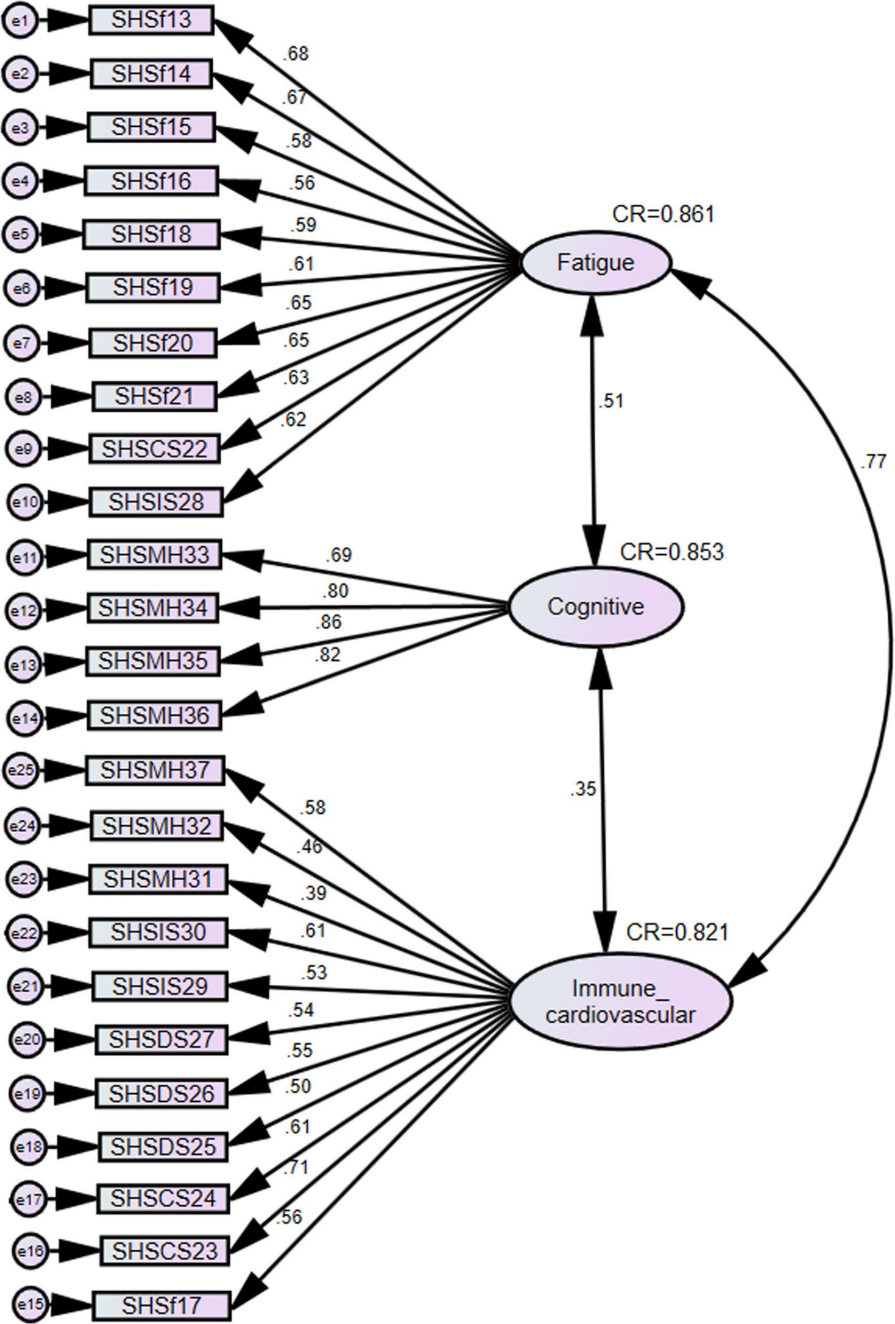
Confirmatory factor model for the three-domain solution. The standardized factor loadings are shown, and the model fit indices are also presented. Suboptimal Health Status (SHS), mental health (MH), cardiovascular system (CS), fatigue (F), immune system (IS)

Using specification search, several candidate models were explored to establish the best paths for the proposed three-factor structure. We further improved the fit by constraining some parameters. The standardized factor loadings for the best model fit for the three-factor structure are presented in Fig. 4. The CFA for the three-factor model recorded very good fit indices, ${\text{RMSEA}} = 0.049 < 0.08\,(90\% \,CI:0.041,0.056),\,{\text{PCLOSE}} = 0.034,\,{\text{CFI}} = 0.903 > 0.9,{\text{GFI}} = 0.880 < 0.9,\,~{\text{TLI}} = 0.907 > 0.9,\,{\text{SRMR}} = 0.055$. All the regression weights for the items are statistically significant (*p* < 0.001) (see Table 3). The composite reliability (CR) statistics indicate construct validity as they are all above the 0.7 threshold. In terms of convergent and discriminant validity, the average variance extract (AVE) was smaller than the maximum shared variance (MSV) for fatigue (AVE = 0.366, MSV = 0.701) and cognitive (AVE = 0.358, MSV = 0.671). The average variance extract (AVE) was greater than the maximum shared variance (MSV) for immune-cardiovascular (AVE = 0.537, MSV = 0.185).

**Table 3:** Regression weights for the fit of the three-domain structural model

Label		Domain	Estimate	Standard error	Critical ratio	*p *value
SHSf17	←	Immune cardiovascular	.924	.127	7.267	***
SHSCS23	←	Immune cardiovascular	.956	.114	8.400	***
SHSCS24	←	Immune cardiovascular	.987	.133	7.435	***
SHSDS25	←	Immune cardiovascular	.599	.091	6.548	***
SHSDS26	←	Immune cardiovascular	.859	.119	7.213	***
SHSDS27	←	Immune cardiovascular	.732	.106	6.929	***
SHSIS29	←	Immune cardiovascular	.782	.114	6.862	***
SHSIS30	←←	Immune cardiovascular	.881	.118	7.457	***
SHSMH32	←	Immune cardiovascular	.690	.108	6.409	***
SHSMH37	←	Immune cardiovascular	1.000			
SHSMH33	←	Cognitive	1.212	.104	11.683	***
SHSMH34	←	Cognitive	.913	.066	13.889	***
SHSMH35	←	Cognitive	1.072	.071	15.108	***
SHSMH36	←	Cognitive	1.000			
SHSf13	←	Fatigue	1.693	.178	9.502	***
SHSf14	←	Fatigue	1.186	.127	9.356	***
SHSf15	←	Fatigue	1.082	.125	8.665	***
SHSf16	←	Fatigue	1.280	.167	7.657	***
SHSf18	←	Fatigue	1.208	.152	7.963	***
SHSf19	←	Fatigue	1.279	.156	8.176	***
SHSf20	←	Fatigue	1.328	.153	8.657	***
SHSf21	←	Fatigue	1.272	.152	8.359	***
SHSCS22	←	Fatigue	.823	.099	8.294	***
SHSIS28	←	Fatigue	1.000			
SHSMH31	←	Immune cardiovascular	.556	.103	5.401	***
Health status	←	Cognitive	.068	.031	2.220	.026
Health status	←	Fatigue	.412	.076	5.451	***
Health status	←	Immune cardiovascular	.286	.057	5.042	***

***Indicates that values are significant at *p* value < 0.001

### Invariance analysis

A multi-group analysis was performed to assess whether the three factors from the CFA are invariant across gender, age and marital status. Gender was categorised as male (n = 96) or female (n = 167), age was treated as a binary variable, with the dataset divided into those below (n = 123) or above (n = 134), mean of 51. Marital status was also treated as a binary variable, with the dataset split into married (n = 168) and not married (n = 89).

Table 4 shows the fit for the multi-group analyses. Constrained models were compared to a baseline model where no constrains were placed on any aspect of the three-factor structural model across multi-groups. Across age, the three-factor model was invariant when the factor loadings are constrained, structural covariance loadings are constrained, and residual covariance loadings constrained (*p* > 0.05). Across marital status, the three-factor model was invariant when the factor loadings and structural covariance loadings were constrained (*p* > 0.05), however, invariance was not achieved when the residual covariance loadings were constrained (*p* = *p* < 0.001). Across gender, the three-factor model was not invariant for any level constrained model (*p* < 0.05).

**Table 4:** Multi-group analysis of fit indices by gender, age group and marital status for three-factor unconstrained model, and models constrained on factor loadings, structural covariance loadings and residual covariance loadings

Model	$\chi$^2^	*df*	*p *value	RMSEA	90% CI	SRMR	CFI	GFI	TLI
*Unconstrained*									
Across gender	742.385	526	–	0.072	[0.063, 0.087]	0.078	0.912	0.830	0.900
Across age group	778.907	526	–	0.078	[0.067, 0.090]	0.077	0.898	0.824	0.883
Across marital status	739.473	526	–	0.073	[0.063, 0.086]	0.068	0.912	0.828	0.899
*Measurement weights*									
Across gender	781.238	548	0.015	0.072	[0.034, 0.087]	0.084	0.896	0.821	0.896
Across age group	806.307	548	0.196	0.078	[0.067, 0.089]	0.078	0.895	0.819	0.885
Across marital status	759.372	548	0.589	0.070	[0.062, 0.085]	0.069	0.912	0.824	0.904
*Structural covariance*									
Across gender	793.156	554	0.005	0.072	[0.065, 0.087]	0.087	0.895	0.819	0.895
Across age group	810.555	554	0.289	0.078	[0.066, 0.089]	0.086	0.896	0.818	0.887
Across marital status	767.887	554	0.443	0.070	[0.062, 0.085]	0.078	0.911	0.823	0.904
*Measurement residuals*									
Across gender	870.873	588	< 0.001	0.073	[0.067, 0.089]	0.091	0.883	0.805	0.883
Across age group	859.787	588	0.054	0.078	[0.066, 0.089]	0.087	0.890	0.811	0.888
Across marital status	841.491	588	< 0.001	0.073	[0.065, 0.087]	0.075	0.895	0.806	0.893

*df* degrees of freedom, *RMSEA* root mean square error of approximation, *CI* confidence interval, *SRMR* standardized root mean square residual, *CFI* comparative fit index, *GFI* goodness-of-fit index, *TLI* Tucker–Lewis Index

## Discussion

The increasing interest in chronic disease prevention has fuelled a predilection for early intervention programmes and early detection instruments. The success of these tools largely depends on the robustness of the instrument, and to a lesser extent, the ease of completing it. The present study describes the psychometric properties of the SHSQ-25 in a Ghanaian population. In the construct validity assessment, we conducted confirmatory factor analysis on the five health subscales of the SHS-Q25 (Fig. 1). It was shown that the five health domains of the SHSQ-25 had moderate-good internal consistency and reliability. After conducting confirmatory factor analysis (CFA), the results revealed that the fit indices for the three-domain model were statistically superior to the five-domain model (Fig. 4). In the CFA, we have included a measurable outcome that may be linked to any “real-world” criteria. That said, the model being fit can only be assessed using the discrepancy between model implied covariances and the observed covariances. Thus, using the χ^2^ test is the obvious statistical test of fit. An alternative strategy is to include some criterion variables external to the SEM analysis, like “clinical or objective data” to examine with regard to their predictive accuracy against some real-world criteria for “Suboptimal Health Status-SHS”. We must admit that the challenge for CFA is that all solutions are entirely “inward facing”. We have performed analysis that is the kind of model or factor structure that contextually suite the population of interest and highlighted substantive explanatory and measurable outcomes dictating the process, or a model evaluated directly using external criterion variables.

A parallel analysis revealed that a three-domain structure was adequate (Fig. 3). A confirmatory factor analysis (CFA) revealed that the fit indices for the three-domain model were statistically superior to the five-domain model (Fig. 4).

Clearly, there is an overlap of the subscales of the SHS-Q25 in the Ghanaian population and the resulting three-factor structure is resigned as fatigue, immune-cardiovascular and cognition. The findings are consistent with the results of our previous study among Chinese that reported χ^2^ (400) = 2517.41, *p* < 0.001 (6); RMSEA = 0.044 (95% CI 0.042 to 0.045), GFI = 0.914 and an overall Cronbach’s $\alpha$ of 0.93. It can be argued that the exploratory structural equation modelling (ESEM), a method that integrates CFA and EFA is thorough and rigorous [23]. The specification search employed allowed the search and comparison of the fit of the models (using indicators such as Akaike information criterion and Bayesian Information Criterion) from several combinations of paths which establishes the thoroughness and robustness of the approach. It is important to note that the inclusion of correlated residuals should not be seen as amounting to model misspecification and must be seen as necessary and legitimate parameters. This is because covariance between exogenous variables or between the errors of endogenous variables reflect non-causal connections and may represent a “don’t-know-situation”. Such models indicate that, in practice, there is the need for the construction of research designs that are sufficiently sophisticated to allow all of the completely anticipated correlated residuals [24].

Moreover, the present study reports low internal consistencies of the immune (0.553) and digestive systems (0.602) when compared with what was reported in our previous study [6]. However, in the Ghanaian context, a compelling reason for this may be due to language translation errors. The literature pinpoints that harmonisation of language is the cornerstone for cross-national comparability [25]. In this study, a significant number of the participants lacked knowledge in the English language used in the questionnaire, thus warranting the need for translation from English to the local Ghanaian language. Although we attempted to ameliorate this by applying forward and reverse translation approaches, the meaning of the questions may have been lost during the translation process. We could have also used a machine translation device but unfortunately, this instrument was not available at the time of data collection.

Meanwhile, the overlap between the results of this study and that of the Chinese could be due to certain intrinsic similarities between the two nations [26]. Like the Chinese economy, Ghana has also seen a tremendous growth in the last few decades, and this reflected in the significant positive changes in macroeconomic indicators including gross domestic product (GDP), consumer price index, stock market prices, industrial production, amongst others. This dramatic growth has paralleled globalisation, affluence and a relentless pace of industrialisation that has triggered sedentary lifestyles, physical inactivity and a quotidian appetite for more westernised diets [12, 14, 20].

Many of these factors, if not all, are stimulus for the incidence of multiple noncommunicable diseases (NCDs). Presently, NCDs account for the death of up to 43% of people, with 19% dying from cardiovascular diseases, 5% from cancers, 2% from chronic respiratory diseases, 3% from diabetes [20]. However, the long latency period for these chronic diseases, coupled with limited health care resources, make it difficult to intervene in a timely fashion. Even when diagnosed, the cost associated with the treatment and management make it difficult to manage the symptoms and live to their full potential. That is why a robust instrument, such as the SHSQ-25 is an invaluable asset not only for the Chinese population but also for Ghanaians. A product of persistent conceptualisation, rigorous testing and evaluation, the SHSQ-25 is user-friendly, can be self-administered or can be completed with minimal assistance from health professional. Once completed, at-risk individuals can be identified for therapies that can prevent or at least delay the onset of these diseases. An effective way to combating chronic disease is to recognise underlying factors and the SHSQ-25 can facilitate that. Currently, the SHSQ-25 is being trialled to predict the onset of chronic disease in 50,000 Chinese individuals.

The present study also shows that a significant number of participants experienced fatigue. This finding is plausible in the light of the literature that suggests urban dwellers including residents of Kumasi are confronted with daily life choices that leaves them with psychological and physiological distress. These include exertion from strenuous activities, work related problems and pressures, inadequate sleep, stress or an underlying medical condition. More intriguing is the statistically significant correlation between fatigue and the immune system (Fig. 4). Research has shown the bidirectional relationship between immune system and the brain [27, 28]. Inflammatory cytokines migrate through neural, humoral and cellular pathways to reach the brain where they interact with the cytokine network. The consequence of this interaction is the activation of the hypothalamic pituitary-adrenal axis (HPA) and the symptoms collectively called the sickness behaviour [27, 28]. This eventually manifests as altered sleep patterns and decreased appetite [27, 28]. Furthermore, our investigation also revealed the association between fatigue and the cardiovascular system. Peckerman indicated a negative correlation between chronic fatigue syndrome and cardiac output [29]. Nelesen et al. [30] found a negative relationship between fatigue and cardiac index and stroke index. However, the study could not find any relationship between fatigue and cardiovascular markers such as blood pressure and heart rate [30]. Another important corollary from the study was that there is an association between the immune system and cardiovascular system. At the cellular level, it has been showed that the heart is interspersed with immune cells including macrophages, dendritic cells and mast cells where they interact with cardiomyocytes, perform housekeeping tasks and involved in cardiac remodelling [31].

The discussion on the robustness of the SHS-Q25 can be ongoing, but we need to highlight some limitations. Firstly, there is an overrepresentation of females which may have introduced some bias in the invariance analysis across gender. Secondly, the discriminant and convergent validity did not provide the desired results as anticipated. However, the three-factor was invariant across age, which is an important risk factor for diabetes. Invariance was achieved for all constrained aspects of the structural model, which establishes the consistency of the instrument. Lastly, the SHSQ-25 is only a subjective instrument and does not provide any objective information. Our previous studies have revealed the association between SHS and objective markers of chronic diseases including the increases of blood pressure, low-density lipoproteins plasma cortisol and mRNA expression of glucocorticoid receptor α/β in lymphocyte [10] and blood glucose [9, 18, 19], endothelial dysfunction [7], and also pregnancy-related disorders [15]. Further, the prevalence of SHS was found to be associated with socioeconomic status, level of education, physical activity, salt intake, smoked or quit smoking, physical activity and adequate dietary intake [14, 20, 22].

Going forward, our research will seek to unravel and discern the link between SHS and objective biomarkers of dysfunctional immune, mental, cardiovascular and the digestive systems. Integrating these markers in SHS research can help to decipher the molecular underpinnings of chronic diseases.

### Implications for clinical practice

The increasing importance of early detection and prevention of chronic disease in the world means that reliable, economical, and easily comprehensible tools such as SHS-25 is required for populations at risk of developing chronic conditions. It is even more important for poor countries with low literacy rate to have access to simple and validated tool for clinical assessment. Thus, investigating the psychometric properties of the SHS-25 tool among the Ghanaian population will provide a reliable and validated tool for healthcare workers to easily assess, detect or prevent the development of chronic conditions in at risk individuals. Given the reliability and the validity of the instrument, healthcare providers can now have confidence in using it to assess the risk of developing chronic conditions among adult population.

## Conclusion

Our study presented aspects of the construct validity of the SHS-Q25 subscales in a Ghanaian population. The fitted models of the SHSQ-25 showed adequate internal consistency and validity in a Ghanaian cohort. The findings in this study suggest the importance of assessing the psychometric properties of the instrument in different samples and encourage future cross-cultural studies. Further, the instrument might be recommended as a screening tool to early detect chronic diseases in resource limited countries such as Ghana.

## Data Availability

The data that support the findings of this study are available from Edith Cowan University, but restrictions apply to the availability of these data, which were used under license for the current study, and so are not publicly available. Data are however available from the authors upon reasonable request and with permission of Edith Cowan University.

## Abbreviations

SHS: Suboptimal Health Status
SHSQ-25: Suboptimal Health Status Questionnaire
NCD: Non-communicable disease
CFA: Confirmatory factor analysis
AVE: Average variance extracts
MSV: Maximum shared variance
SEM: Structural equation model
SBP: Systolic blood pressure
DBP: Diastolic blood pressure
BMI: Body mass index
FPG: Fasting plasma glucose
KMO: Kaiser–Meyer–Olkin
EFA: Exploratory factor analysis
RMSEA: Root mean square error of approximation
GFI: Goodness-of-fit index
TLI: Tucker–Lewis Index
CR: Composite reliability
WHtR: Waist-to-height ratio
